# Modeling biomass allocation strategy of young planted *Zelkova serrata* trees in Taiwan with component ratio method and seemingly unrelated regressions

**DOI:** 10.1038/s41598-021-87129-7

**Published:** 2021-04-06

**Authors:** Chieh-Yin Chen, Shu-Hui Ko, Tzeng Yih Lam

**Affiliations:** 1 The Experimental Forest, College of Bioresources and Agriculture, National Taiwan University, No. 12, Sec. 1, Qianshan Rd., Zhushan Township, 55750 Nantou County Taiwan; 2 Taiwan Forestry Research Institute, No. 53, Nanhai Road, Taipei, 10066 Taiwan; 3 School of Forestry and Resource Conservation, National Taiwan University, No. 1, Sec. 4, Roosevelt Rd., Taipei, 10617 Taiwan

**Keywords:** Plant sciences, Forestry, Ecological modelling, Statistical methods

## Abstract

Trees accumulate biomass by sequestrating atmospheric carbon and allocate it to different tree components. A biomass component ratio is the ratio of biomass in a tree component to total tree biomass. Modeling the ratios for *Zelkova serrata*, an important native reforestation tree species in Taiwan, helps in understanding its biomass allocation strategy to design effective silvicultural treatments. In this study, we applied Component Ratio Method (CRM) to relate biomass component ratios of main stem, large branch, twig, and foliage to tree attributes of *Z*. *serrata* from a 9-year-old plantation. Nonlinear and linear CRM models were fitted with Seemingly Unrelated Regression to account for model correlations. Linear CRM models with dbh as the predictor had the best fit with model correlations as high as 80%. About 46% and 40% of total tree biomass was allocated to main stem and large branch, respectively. However, main stem biomass decreased by 1.9% with every 1-cm increase in dbh, but large branch biomass increased by 2.2% instead. Results suggest that dominant *Z. serrata* trees tend to branch and fork, while smaller trees invest in larger main stem. An early pruning treatment should focus on dominant trees to maintain crown ratio and ensure wood quality.

## Introduction

A unique contribution of trees to ecosystem services beneficial to human society is the accumulation of carbon in the form of biomass. As such, the Paris Agreement formally recognizes that forests play an important role in addressing the impact of climate change by sequestrating carbon from atmosphere^1^. As the world is moving towards decarbonization, many mitigation methods have been developed such as negative emissions technologies (NETs)^2^ and radiative forcing geoengineering^3^. Among the different NETs, afforestation and reforestation approach^2^ is immediately relevant to forest management. By planting reclaimed lands or degraded forests, standing trees accumulate biomass throughout their life cycles albeit at different rates. Forests could also contribute to other climate change mitigation methods such as biochar production^4^. Matovic suggested that about 4.8 Gt of carbon could be sequestrated if 10% of the world biomass was converted to biochar, and part of the biomass could be sourced from forest management activities^4^. Osman et al. comprehensively reviewed several decarbonization technologies and found that using plant biomass as fuel in the oxyfuel combustion route could promote bioenergy and carbon capture and storage (BECCS) system as an effective way to achieve decarbonization^5^. Thus, there are many pathways available for forests to assist climate change mitigation.

Tree biomass is not directly measurable and is usually estimated by different methods. The most common approach is first estimating tree volume from forest inventory data and converting it to tree biomass by biomass expansion factors^6,7^. An alternative approach is using allometric equations to estimate tree total biomass or biomass of each tree component from stem diameter at breast height (dbh)^6,8^. Modeling how a tree partitions its total biomass into different tree components is needed. It is because understanding distribution of wood biomass within a tree is important to estimate its wood utilization potential and economic value of tree components^9^. This also helps with designing appropriate silviculture treatments that promote biomass accumulation in certain tree components.

Jenkins et al. proposed a Component Ratio Method (CRM) in which ratio of biomass of a tree component was nonlinearly associated with dbh^10^. A biomass component ratio is calculated as the proportion of biomass of a tree component (e.g., main stem) to total tree aboveground biomass^10^. As a result, ratios of all biomass components should sum up to one. CRM has the advantage of additivity such that predicted biomasses of all the components in a tree sum to the predicted total tree biomass^11,12^. A hybrid approach could be used to estimate tree component biomass: tree total biomass is first predicted from tree volume by a biomass expansion factor, and CRM is then used to consistently distribute this total to each tree component^6^. Thus, CRM could assist in designing silvicultural treatments that maximize wood utilization potential of a whole tree or certain tree components. While CRM is very useful, there are very few studies that model relationship between biomass component ratios and dbh^13,14^.

In Taiwan, forests occupy about 60% of the total land area of about 36,200 km^2^, which could be grouped into five general types: broadleaf forest, coniferous forest, mixed broadleaf-coniferous forest, mixed bamboo broadleaf forest, and mixed bamboo coniferous forest^15,16^. The 4th Taiwan National Forest Resource Inventory conducted between 2008 and 2012 reported that total carbon storage in Taiwan forests was 754.3 Mt CO_2_ or 347.9 t CO_2_ ha^−1^^15,17^. Of the total carbon storage, the largest proportion of carbon is stored in broadleaf forests (468.9 Mt CO_2_) followed by coniferous forests (156.3 Mt CO_2_) and mixed broadleaf-coniferous forests (103.5 Mt CO_2_)^15^. However, on a per unit area basis, mixed broadleaf-coniferous forests store the most amount of carbon (604.7 t CO_2_ ha^−1^) followed by coniferous forests (522.3 t CO_2_ ha^−1^) and broadleaf forests (319.1 t CO_2_ ha^−1^)^15^. *Zelkova serrata* (Thunb.) Makino is an important reforestation broadleaf tree species native to Taiwan. It distributes from 300 to 1000 m a.s.l. and has high economic value due to its desirable wood properties for construction, furniture, flooring, and sculpture^18^. As such, *Z*. *serrata* is one of the ten major tree species widely planted since the start of the Taiwan National Reforestation Program in 1996^19^. A total of 1785 ha of land has been reforested with the tree species between 1997 and 1999^19^. Because of its importance as a plantation tree species, there is a continuing interest in understanding its role in carbon sequestration. The 4th Taiwan National Forest Resource Inventory reported that *Z*. *serrata* plantation had an average stand volume of 193.2 m^3^ ha^−1^, which stored about 452.2 t CO_2_ ha^−1^—higher than the average storage rate of broadleaf forests^15,17^. A 9-year-old *Z. serrata* plantation could hold up to 267.9 t CO_2_ ha^−1^^18^. Depending on stand age, annual stand-level carbon sequestration rate could be between 1.81 to 4.11 t CO_2_ ha^−1^ year^−1^^18,20^. The biomass expansion factors for a 25-year-old and a 46-year-old *Z. serrata* plantations were estimated to be 1.328 Mg m^-3^ and 1.528 Mg m^-3^, respectively^20^. Lastly, CO_2_ fixation rates of the upper-leaf and lower-leaf of *Z. serrata* species were estimated to be 5.52 g m^-2^ s^−1^ and 2.38 g m^-2^ s^−1^, respectively^21^.

While past studies have assessed carbon sequestration potential and biomass of *Z. serrata* on a stand-level, they have not explored the strategy adopted by the tree species in distributing its total biomass among its various components. As mentioned above, understanding this allocation strategy has ecological, economic, and management implications. Thus, to fill in the knowledge gap, the goals of this study were to apply CRM to model tree-level relationship between proportion of biomass in each tree component and dbh of *Z. serrata*, and to suggest potential silvicultural treatments that improve wood utilization potential of the tree species.

## Materials and methods

### Study site

The study site was established at a *Z*. *serrata* plantation in the Neimaopu Forest District of the National Taiwan University Experimental Forest. The plantation was established in 1997 with a planting density of 1500 trees/ha and an area of 1.3 ha. It was located at 23° 40′ N and 120° 50′ E at 800 m a.s.l. Mean annual precipitation in the area was 1853 mm between 1997 and 2004. Mean annual temperature was 21.5 °C with mean relative humidity of 81.4%. This study was carried out in 2005 when the planted *Z*. *serrata* trees were 9 years old. All trees in the 1.3 ha plantation were censused for dbh and tree height (ht). A total of 921 trees were measured. The sampled trees were grouped into five diameter classes of 5-cm width (Table 1). A total of 12 trees were randomly selected from the first four diameter classes for biomass study with two trees from the diameter class of ≤ 5 cm, four trees from the diameter class of 5–10 cm, and three trees from each of the diameter classes of 10–15 and 15–20 cm (Table 1). Since the last diameter class has only 3 sampled trees, no tree was selected from it. Thus, the 12 selected trees represented the range of tree attributes in the study site.

**Table 1:** Number of census trees and sampled trees for biomass study in each diameter class.

Diameter class (cm)	Number of census trees	Number of sampled trees for biomass	Dbh (cm)	Height (m)
≤ 5	167	2	4.43 (18.4%)	5.85 (18.9%)
5–10	518	4	8.28 (6.6%)	6.61 (15.9%)
10–15	208	3	13.37 (9.8%)	9.46 (15.1%)
15–20	25	3	18.63 (5.9%)	9.23 (26.0%)
> 20	3	–	–	–

Mean diameter at breast height (dbh) and mean tree height of the trees sampled for biomass study with their respective coefficient of variation (%) in brackets are reported for each diameter class.

### Biomass sampling protocol

For a sampled *Z*. *serrata* tree, the tree was felled at the base and separated into four components in the field: main stem, large branches, twigs, and foliage. Fresh weight (kg) of each component was measured. Stem analysis was carried out for the main stem. The main stem was separated into 1-m sections. A stem disc was collected at the top of each section, and its fresh weight (kg) was measured. Large branches, twigs, and foliage were subsampled, and the samples of the three components were measured for their fresh weights (kg). All stem discs and samples were brought back to laboratory and dried at 65 °C until constant weight. The oven-dried stem discs and samples of large branches, twigs, and foliage were measured for their dried weights (kg). A ratio of dried weight to fresh weight for each component (i.e., stem, large branch, twig, and foliage) was calculated from the samples. For each component, the ratio was applied to convert the fresh weight of the component recorded in the field to its dried weight biomass (kg).

### Statistical analysis

The above ground biomass (AGB, kg) of a sampled *Z*. *serrata* tree was defined as the sum of its four component dried weight biomasses: stem biomass (B_stem_, kg), branch biomass (B_branch_, kg), twig biomass (B_twig_, kg), and foliage biomass (B_foliage_, kg). For each sampled tree, stem biomass ratio (R_stem_ = B_stem_/AGB), branch biomass ratio (R_branch_ = B_branch_/AGB), twig biomass ratio (R_twig_ = B_twig_/AGB), and foliage biomass ratio (R_foliage_ = B_foliage_/AGB) were calculated with the four ratios summed to one. The four ratios were used to build the CRM for each biomass component. Two biomass ratio models were applied based on a nonlinear model^10^ (Eq. 1) and a linear model (Eq. 2) relating a biomass component ratio to a tree attribute,

1 $$R_{c} = \exp \left( {\beta_{0} + \frac{{\beta_{1} }}{X}} \right)$$

2 $$R_{c} = \beta_{0} + \beta_{1} X$$

where, *c* denoted a biomass component, and *X* was a predictor. Three predictors were considered: dbh, dbh^2^, and dbh^2^·ht. The first predictor was the tree diameter, the second predictor represented tree basal area, and the third predictor represented tree volume. As a result, there were a total of six combinations of model and predictor for developing the *Z*. *serrata* CRM.

For R_foliage_, preliminary data analysis and model fitting showed that the parameter *β*_*1*_ was not statistically significantly different from zero for the six combinations of model and predictor. This suggested that foliage biomass was not significantly associated with dbh, tree basal area, and tree volume. Hence, following the suggestions by Jenkins et al. and Radtke et al., the six combinations of model and predictor were only fitted to R_stem_, R_branch_, and R_twig_^10,13^. As the four ratios should sum to one, R_foliage_ was calculated by subtracting the sum of the other three component ratios from one, i.e., R_foliage_ = 1 – (R_stem_ + R_branch_ + R_twig_). As a result, the three component ratio equations (i.e., R_stem_, R_branch_, and R_twig_) was integrated as a system for each combination of model and predictor. To properly develop such a system, one should consider that the component ratios were dependent and the residuals were correlated because the same tree gave the values to the three component ratios^22^. To account for potentially correlated residuals, Seemingly Unrelated Regression (SUR^12,23^) was used to fit a system of the three component ratio equations for each of the six combinations of model and predictor. In particular, Nonlinear Seemingly Unrelated Regression (NSUR) was applied to Eq. (1), and Linear Seemingly Unrelated Regression (LSUR) was applied to Eq. (2). Comparison between the combinations was made by examining residual standard error (RSE) and residual plots. The best system for the three component ratio equations was chosen. All analyses were carried out in R using systemfit package^24,25^.

### Ethics declarations/protocol compliance

The experimental and field protocols of collecting plant materials in this study were performed in accordance with relevant institutional and national guidelines and regulations.

## Results

### Model selection

**Figure 1:**
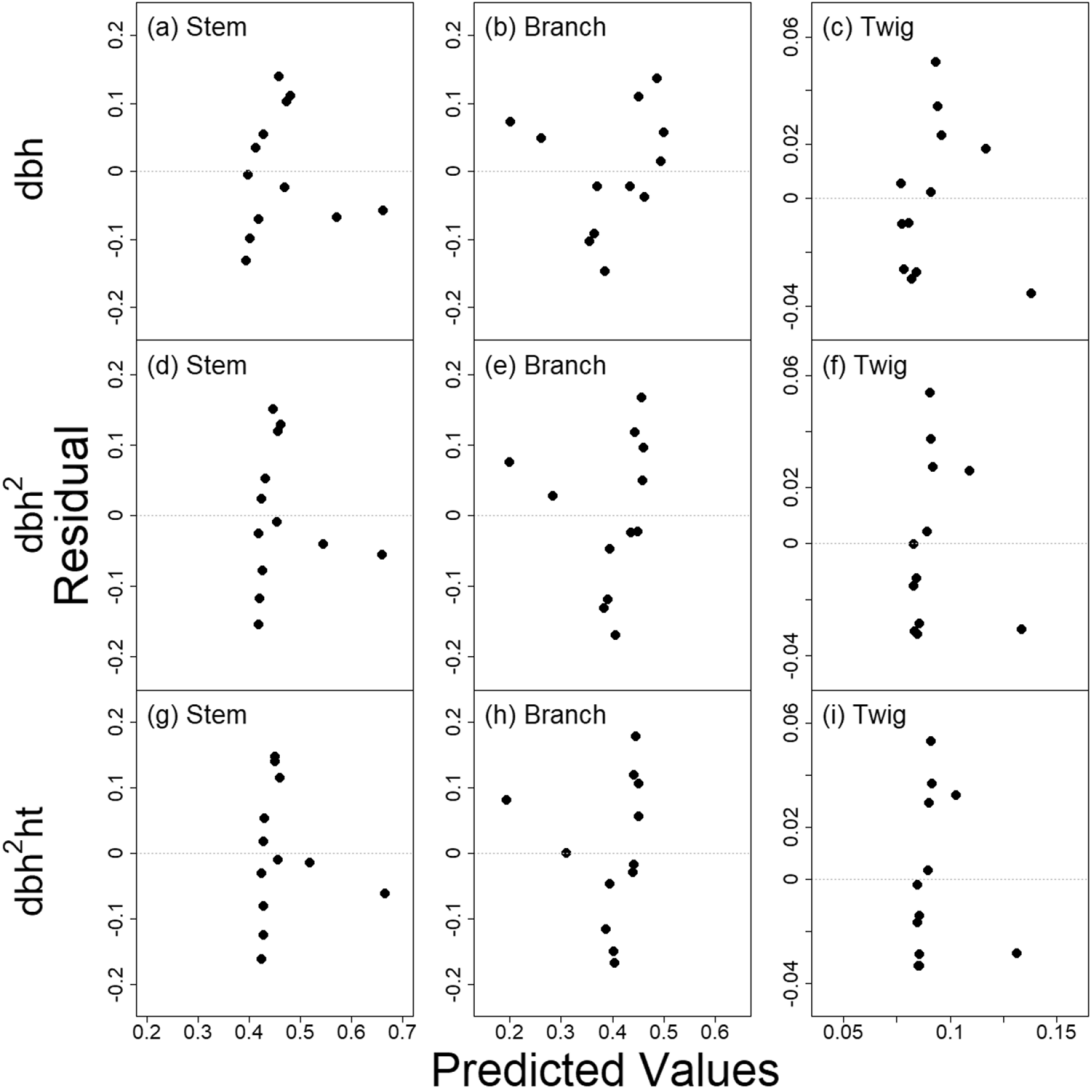
Residuals vs. predicted values of three Nonlinear Seemingly Unrelated Regression models (Eq. 1). The three predictors are: **(a–c)** dbh, **(d–f)** dbh^2^, and **(g–i)** dbh^2^·ht. Each NSUR model consists of three Component Ratio Models (CRM) for the three biomass components: **(a,d,g)** main stem, **(b,e,h)** large branch, and **(c,f,i)** twig.

There was a total of six combinations of two SUR models and three predictors fitted to the *Z*. *serrata* dataset to build the CRM for the three biomass components. Selecting the final *Z*. *serrata* CRM model was based on: (1) residual plots, (2) comparisons of RSE, and (3) levels of significance of the estimated parameter *β*_*1*_ in the fitted CRMs. Residual plots of the three NSUR models for the three predictors (i.e., dbh, dbh^2^, and dbh^2^·ht) depicted clustering of residuals over a small range of predicted values (Fig. 1). The clustering of residuals was especially prominent for the NSUR models with dbh^2^ and dbh^2^·ht (Fig. 1d–i). For example, for the NSUR model with dbh^2^·ht, the residuals ranged from − 0.15 to 0.15% for a predicted value of about 0.43 for R_stem_ (Fig. 1g). This implied that predicted values of a component ratio for a tree attribute were very similar even though the actual observed values were different. This could be an issue when predicting a component ratio for a new tree.

**Figure 2:**
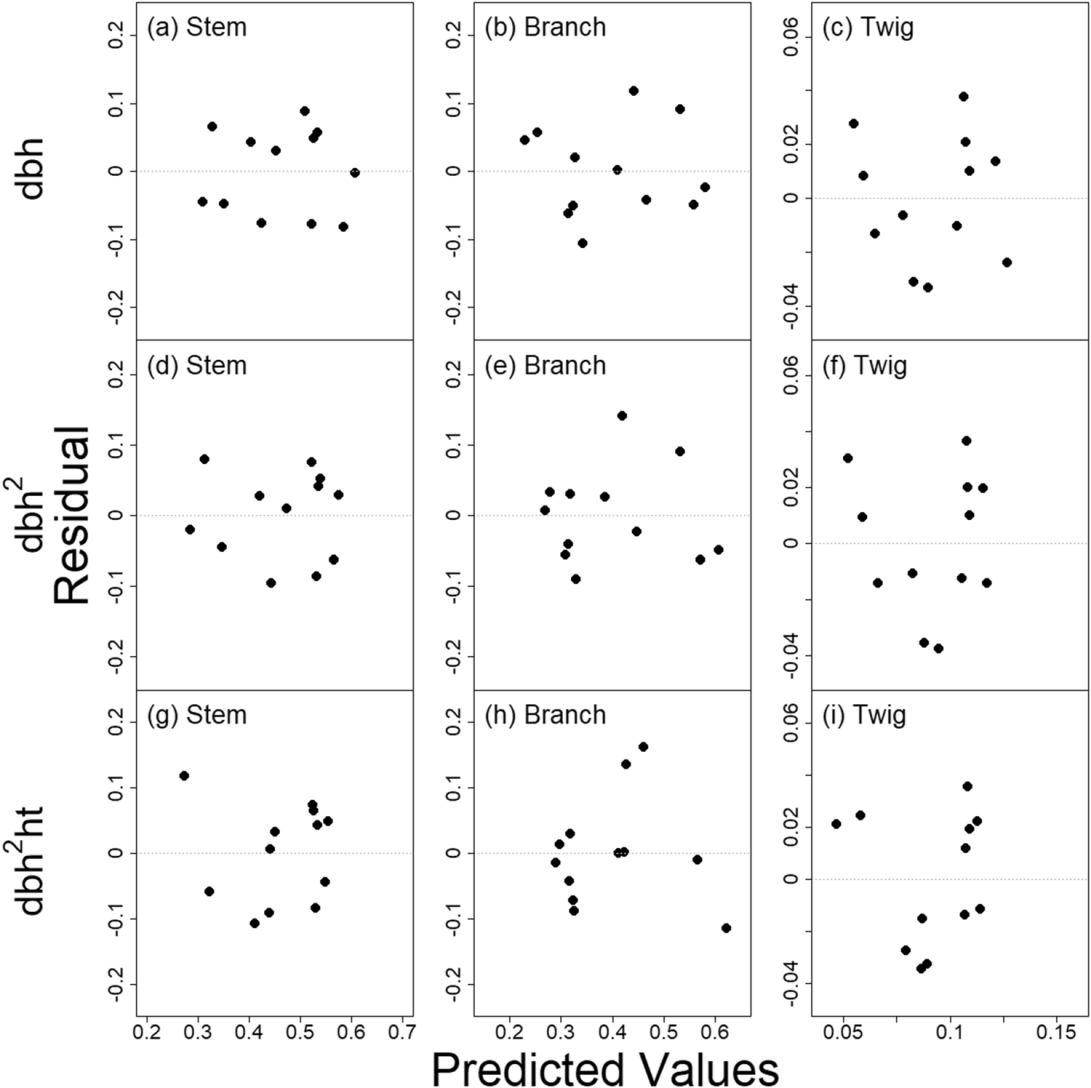
Residuals vs. predicted values of three Linear Seemingly Unrelated Regression models (Eq. 2). The three predictors are: **(a–c)** dbh, **(d–f)** dbh^2^, and **(g–i)** dbh^2^·ht. Each LSUR model consists of three component ratio models (CRM) for the three biomass components: **(a,d,g)** main stem, **(b,e,h)** large branch, and **(c,f,i)** twig.

On the contrary, residuals of three LSUR models for the three predictors were more dispersed over the range of predicted values (Fig. 2). Moreover, the range in the residuals of the three LSUR models was smaller than their NSUR counterparts. The LSUR model with dbh generally produced more homogeneously dispersed residuals without an obvious trend across the range of predicted values consistently for the three biomass component ratios (Fig. 2a–c) compared to the residuals from the two LSUR models with dbh^2^ and dbh^2^·ht (Fig. 2d–i).

Agreeing with the residual plots, the RSEs of the three NSUR models were consistently larger than their LSUR counterparts, which could be 20–60% larger depending of the component ratio (Table 2). The discrepancy was particularly large for R_stem_. Moreover, for the two NSUR models with dbh^2^ and dbh^2^·ht, the estimated *β*_*1*_ for the R_twig_ were not significantly different from zero (Table 2). Among the three LSUR models, the LSUR model with dbh generally had the lowest or comparable RSE than the two LSUR models with dbh^2^ and dbh^2^·ht (Table 2). Furthermore, its estimated *β*_*1*_ for the three biomass component ratios were more highly significant than the estimated *β*_*1*_ of the two LSUR models with dbh^2^ and dbh^2^·ht, i.e., smaller *p*-values (Table 2). Considering the consistency across the three assessment criteria, the LSUR model with dbh performed the best and was chosen to build the *Z*. *serrata* CRM system.

**Table 2:** Residual standard errors of fitted component ratio method models for the biomass components and for the combination of models and predictors.

Predictor × model	Biomass component		
	Stem	Branch	Twig
**Dbh**			
Nonlinear	0.0934/***	0.0923/***	0.0290/*
Linear	0.0658/***	0.0711/***	0.0243/***
**dbh**^**2**^			
Nonlinear	0.1029/**	0.1109/**	0.0315/ns
Linear	0.0640/***	0.0716/***	0.0258/*
**dbh**^**2**^**·ht**			
Nonlinear	0.1054/**	0.1157/**	0.0323/ns
Linear	0.0784/**	0.0858/**	0.0263/*

The biomass components are main stem, large branch, and twig. The two models are nonlinear and linear SUR. The three predictors are dbh, dbh^2^, and dbh^2^·ht. RSE is residual standard error. The *p*-values of the estimated parameter *β*_*1*_ in the fitted Component Ratio Method models (Eqs. 1 and 2) are represented by asterisk next to the reported RSEs: ns (*p* > 0.05), * (*p* ≤ 0.05), ** (*p* ≤ 0.01), *** (*p* ≤ 0.001), **** (*p* ≤ 0.0001).

### Component ratio model (CRM)

The largest biomass component ratio of the sampled *Z. serrata* trees was R_stem_ with an average and standard deviation of 0.463 ± 0.118 (range = 0.264 to 0.604). The second largest biomass component ratio was R_branch_ with an average and standard deviation of 0.399 ± 0.136 (range = 0.237 to 0.624). R_twig_ had an average and standard deviation of 0.092 ± 0.033 (range = 0.052 to 0.144). R_foliage_ was the smallest with an average and standard deviation of 0.046 ± 0.023 (range = 0.018 to 0.096). Despite obvious difference in the average values, the range of the four tree component ratios was fairly wide. Especially, the range showed large overlapping between R_stem_ and R_branch_, and between R_twig_ and R_foliage_.

The fitted LSUR model had an overall *R*^*2*^ of 0.73 suggesting that the model overall goodness of fit was good with about 73% of the total variance in biomass component ratios linearly explained by dbh. Fitted LSUR suggested a very high negative correlation between R_stem_ and R_branch_ linear models (− 0.834; Table 3), but a more moderate negative correlation between R_branch_ and R_twig_ linear models (− 0.341; Table 3). Thus, the moderate to high correlation between two component ratio models highlighted the need to apply SUR in model fitting.

**Table 3:** Estimated parameters and properties of the final fitted linear seemingly unrelated regression models (Eq. 2)**.**

Fitted component ratio models			
	Estimate	Standard error	*p*-value
**Stem**			
*β*_*0*_	0.6790	0.0468	< 0.0001
*β*_*1*_	− 0.0188	0.0037	0.0005
RSE	0.0658		
Multiple R^2^	0.7172		
**Branch**			
*β*_*0*_	0.1437	0.0506	0.0176
*β*_*1*_	0.0222	0.0040	0.0003
RSE	0.0711		
Multiple R^2^	0.7525		
**Twig**			
*β*_*0*_	0.1441	0.0173	< 0.0001
*β*_*1*_	− 0.0045	0.0014	0.0082
RSE	0.0243		
Multiple R^2^	0.5194		

Correlation between component ratio models			
	Stem	Branch	Twig
Stem	1.0000	− 0.8340	− 0.1400
Branch	− 0.8340	1.0000	− 0.3407
Twig	− 0.1400	− 0.3407	1.0000

The estimated parameters are reported for the main stem, large branch, and twig biomass component ratios. RSE is residual standard error.

**Figure 3:**
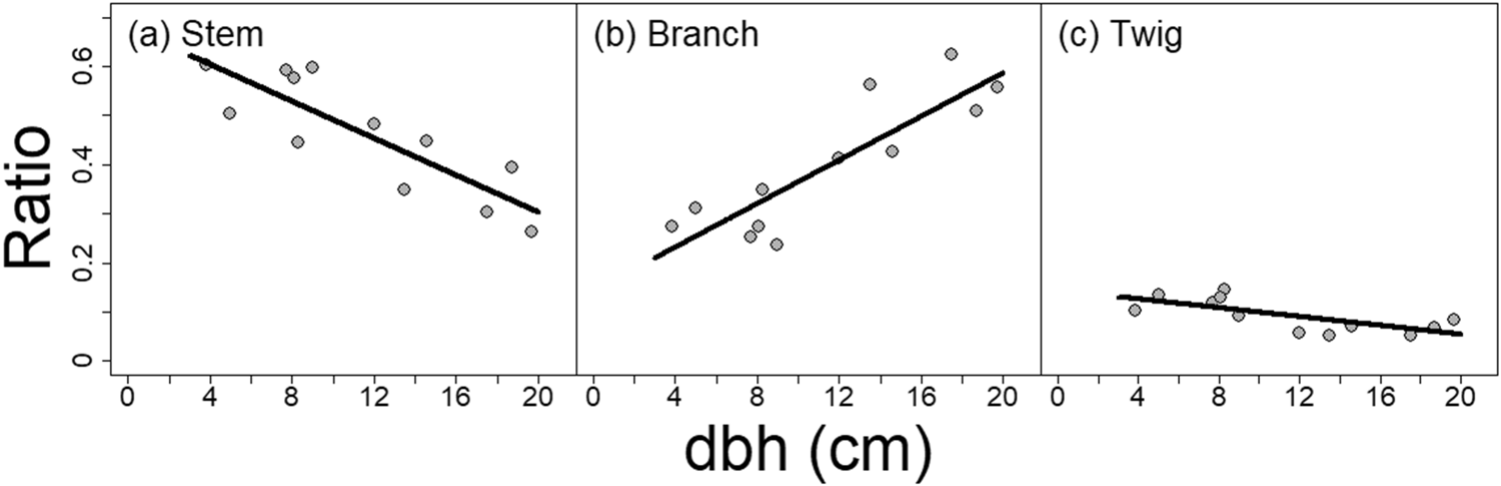
Final fitted Linear Seemingly Unrelated Regression models (Eq. 2) of the component ratios over dbh. The biomass component ratios are: **(a)** main stem, **(b)** large branch, and **(c)** twig. Black solid lines depict fitted regression models. Gray circles depict observed ratios.

In general, dbh explained the variance of each biomass component ratio relatively well with multiple *R*^*2*^ ranging from 0.52 to 0.75 (Table 3). However, the linear relationship between dbh and R_stem_, R_branch_, and R_twig_ was different. For R_stem_, the relationship was negative with an increase of 1 cm in dbh correlated with a decrease of 0.019 in R_stem_ (*p*-value = 0.0005; Fig. 3a, Table 3). On the other hand, for R_branch_, the relationship was positive with an increase of 1 cm in dbh correlated with an increase of 0.022 in R_branch_ (*p*-value = 0.0003; Fig. 3b, Table 3). Lastly, for R_twig_, the relationship was negative with an increase of 1 cm in dbh correlated with a decrease of 0.0045 in R_twig_ (*p*-value = 0.0082; Fig. 3c, Table 3).

## Discussion

Many past studies focused on modeling the relationship between dry weight biomass of tree components and tree attributes^26,27^. However, very few studies have modeled the relationship with ratio of biomass in each tree component to AGB^10,13^. Woodall et al. applied the CRM models^10^ to estimate biomass and carbon content of trees in USA using the national forest inventory data. Our study contributes to the continuing modeling efforts to understand relationship between biomass component ratios and tree attributes. Our study is also unique in that it is the first to model CRM under SUR framework anticipating that there would be correlation between models. This has been supported in the results with correlation as high as 80%. Carvalho and Parresol suggested that it would be more realistic to consider component biomasses being dependent and residuals being correlated^28^. SUR should lower estimated variances of regression parameters, which means higher efficiency in estimating parameters and producing reliable prediction intervals^11,12^. Thus, results would be more reliably interpreted when applying SUR, and would in turns lead to more confidence in decision making such as designing effective silvicultural treatments. For that matter, CRM should be analyzed under the SUR framework as would other studies on dried weight biomass^22^.

For studies on biomass component ratio^10,13^ and on dry weight biomass component^6,29^, nonlinear relationship in the form of exponential distribution has been used to relate biomass ratio or biomass to dbh. In contrast, the nonlinear model (Eq. 1) in our study had poor predictability. The residual plots suggested that the fitted nonlinear models predicted similar values of biomass component ratios for trees of different dbhs. On the contrary, the linear model (Eq. 2) had better predictability. A possible explanation that the linear model performed better could be due to small sample size from the young *Z*. *serrata* stand. That being said, the sampled trees covered a wide range in dbh from 3.9 cm to 19.7 cm suggesting the scope of inference for the final LSUR CRM models should be adequate. Hence, multiple model forms should be compared when building a CRM system for a tree species.

Past studies have assessed carbon sequestration potential of *Z. serrata* tree species on a per area basis, e.g., with forest inventory data for Taiwan^30^, with remote sensing data in an urban forest in USA^31^, and for urban forests in South Korea^32,33^. None of these studies has assessed how *Z. serrata* distributing biomass among its components on tree-level. While the sampled trees in our study are of the same age, the *Z. serrata* stand exhibited strong horizontal and vertical stratification with a wide range in dbh and ht. The fitted CRM showed clear tendency of dominant *Z. serrata* trees to allocate biomass into developing larger branches at the expense of main stem biomass. One would expect that investing in larger branches is for crown development. However, the final fitted CRM suggested otherwise as there was no statistically significant increase in foliage biomass in dominant trees. Moreover, twig biomass in dominant trees were less than that in smaller diameter trees. On the contrary, for intermediate or suppressed *Z. serrata* trees, majority of sequestrated carbon is allocated to developing main stem according to the final fitted CRM system. While *Z. serrata* is highly adaptive to grow in a range of environments, thus preferred as a reforestation species, they are prone to branching and forking^34^. In a study^35^, 82% of *Z. serrata* trees in a five-year-old plantation developed forks with 44% of the trees forked at 1.3 m and below while 39% of the trees above 1.3 m. This corresponds with our study in that *Z. serrata* tends to branch when local growing conditions are favorable for it to become dominant. Thus, pruning was necessary to increase wood utilization of *Z. serrata* trees unless seedlings were planted in high density or with genetic selection^34^. This is supported by our study especially for dominant *Z. serrata* trees, which should be pruned early to avoid undesirable wounds.

From an economical perspective, early pruning of dominant trees and planting seedlings in high density both incur additional operational costs. However, economic gain from planting in high density could potentially offset the additional costs of planting materials and labor. It is generally observed that planting seedlings in high density tends to limit individual tree diameter growth due to increase competition^36^. From our modeling results, we speculate that high planting density of *Z. serrata* would lead to greater allocation of biomass to main stem instead of forming large branches, i.e., less forking and branching. This would reduce the cost of pruning at early stand development and increase extraction ratio when the stand is mature for thinning operation. Extraction ratio is defined as the ratio of harvested wood transported out of a forest to total wood harvested^37^. Higher extraction ratio implies greater economic returns from production of wood products, which could also serve as long-term carbon storage or fuel for BECCS through methods such as oxyfuel combustion^5^. However, an in-depth economic study such as net present value analysis is necessary to fully understand the implications on stand- and landscape-level^38,39^. Nevertheless, our study shows that modeling biomass allocation strategy of *Z*. *serrata* would have economical implication for Taiwan forestry as the tree species will continue to be important in reforestation effort.

It would be fairly easy to apply the developed CRM system in our study to assess biomass allocation in a *Z. serrata* plantation with a hybrid approach^6^. For a *Z. serrata* tree in a sample plot, its volume is first estimated and converted to total tree dry weight biomass with the biomass expansion factors from Lin et al^20^. The biomass component ratios of the four tree components (R_stem_, R_branch_, R_twig_, and R_foliage_) are predicted from its dbh accordingly with our fitted LSUR models (Table 3). From which, one could then estimate dried weight biomass of the four tree components of the sample tree, which in turns could be expanded to per area basis with appropriate expansion factors associated with the sample plot^40^.

Despite that our study was carried out in a single even-aged stand, it is the first to suggest that there are significant differences in biomass allocation strategy for *Z. serrata* trees of different sizes at the early stage of stand development. Most of the *Z. serrata* plantations established during the Taiwan National Reforestation Program should be currently about 20 years old. We conjecture that dominant trees that are already in the main canopy in early stand development stage will likely continue the same growth trajectory and biomass allocation strategy, and so would the suppressed trees. However, future study should resample in the same study site, which is now 23 years old, to test our hypothesis.

## Conclusion

This study is the first to model biomass allocation strategy of planted *Z. serrata* trees. It is one of the few studies to model biomass allocation with the CRM approach, and is also the first to model CRM under SUR framework to properly account for correlations between models. Our developed CRM could also be used to approximately predict tree component biomasses of a *Z. serrata* plantation when only carbon estimate per unit area is available. For example, a *Z*. *serrata* plantation stores about 452.2 t CO_2_ ha^−1^^15^. Of this amount, our CRM models suggest that 209.4, 180.4, 41.6, and 20.8 t CO_2_ ha^−1^ are stored in main stem, large branch, twig, and foliage, respectively. Contrary to other studies, our results supported a linear relationship between biomass component ratios and dbh instead of a nonlinear relationship. The fitted linear relationship suggests that *Z. serrata* trees in the main canopy have larger sized crowns because of the tendency in forking and branching, which could more effectively compete for resources and suppress development of the other trees. Pruning is necessary not only to improve wood utilization potential of dominant *Z. serrata* trees by reducing knots but also to allow other trees in a stand to develop their utilization potential. Future work could sample *Z. serrata* trees across stand development stages and elevation to examine whether there is any change in biomass allocation strategy of the tree species under different stand age and growing conditions. Moreover, studying potential effects of various silvicultural treatments on biomass allocation strategy of the tree species and economic tradeoff could lead to better planning of wood utilization in long-term carbon storage or bioenergy production.

## Data Availability

The datasets generated during and/or analyzed during the current study are available from the corresponding author and co-authors on reasonable request.

## References

[1] UNFCCC. *Adoption of the Paris Agreement*. 32 (2015).

[2] Fuss S (2018). Negative emissions—Part 2: Costs, potentials and side effects. Environ. Res. Lett..

[3] Lawrence MG (2018). Evaluating climate geoengineering proposals in the context of the Paris Agreement temperature goals. Nat. Commun..

[4] Matovic D (2011). Biochar as a viable carbon sequestration option: Global and Canadian perspective. Energy.

[5] Osman AI, Hefny M, Abdel Maksoud MIA, Elgarahy AM, Rooney DW (2020). Recent advances in carbon capture storage and utilisation technologies: A review. Environ. Chem. Lett..

[6] Clough BJ (2018). Testing a new component ratio method for predicting total tree aboveground and component biomass for widespread pine and hardwood species of eastern US. Forestry.

[7] Lam TY, Li X, Kim RH, Lee KH, Son YM (2015). Bayesian meta-analysis of regional biomass factors for *Quercus mongolica* forests in South Korea. J. For. Res..

[8] Sileshi GW (2014). A critical review of forest biomass estimation models, common mistakes and corrective measures. For. Ecol. Manag..

[9] Ver Planck, N. R. & MacFarlane, D. W. A vertically integrated whole-tree biomass model. *Trees* **29**, 449–460, 10.1007/s00468-014-1123-x (2015).

[10] Jenkins JC, Chojnacky DC, Heath LS, Birdsey RA (2003). National-scale biomass estimators for United States tree species. For. Sci..

[11] Parresol BR (2001). Additivity of nonlinear biomass equations. Can. J. For. Res..

[12] Parresol, B. R. Assessing tree and stand biomass: A review with examples and critical comparisons. *For. Sci.* **45**, 573–593, 10.1093/forestscience/45.4.573 (1999).

[13] Radtke P (2017). Improved accuracy of aboveground biomass and carbon estimates for live trees in forests of the eastern United States. Forestry.

[14] Woodall, C. W., Heath, L. S., Domke, G. M. & Nichols, M. C. Methods and equations for estimating aboveground volume, biomass, and carbon for trees in the U.S. forest inventory, 2010. **30**, 10.2737/NRS-GTR-88 (2011).

[15] Chiou L-W, Huang C-H, Wu J-C, Hsieh H-R (2015). Report of the 4th National Forest Resource Inventory in Taiwan. Taiwan For. J..

[16] Yang T-R, Lam TY, Kershaw JA (2018). Developing relative stand density index for structurally complex mixed species cypress and pine forests. For. Ecol. Manag..

[17] Taiwan Forestry Bureau. *The Fourth National Forest Resource Inventory*. Vol. 78 (2017).

[18] Ko, S.-H. *Study on the Biomass and Carbon Storage in the Zelkova serrata Plantation*. MSc. Thesis, National Chung-Hsing University, 10.6845/NCHU.2006.00871 (2006).

[19] Lin, J.-C., Jeng, M.-R., Liu, S.-F. & Lee, K. J. Economic benefit evaluation of the potential CO_2_ sequestration by the National Reforestation Program. *Taiwan J. For. Sci.* **17**, 311–321, 10.7075/TJFS.200209.0311 (2002).

[20] Lin K-C, Huang C-M, Duh C-T (2008). Study on estimate of carbon storages and sequestration of planted trees in *Zelkova serrata* plantations, Taiwan. J. Natl. Park.

[21] Liao S-H, Wang Y-N (2002). Study on carbon dioxide fixation efficiency of *Cinnamomum camphora* and *Zelkova serrata* in understory planting. Q. J. Chin. For..

[22] Lambert MC, Ung CH, Raulier F (2005). Canadian national tree aboveground biomass equations. Can. J. For. Res..

[23] Zellner A (1962). An efficient method of estimating seemingly unrelated regressions and tests for aggregation bias. J. Am. Stat. Assoc..

[24] Henningsen, A. & Hamann, J. D. systemfit: A package for estimating systems of simultaneous equations in R. *J. Stat. Softw.* **23**, 1–40, 10.18637/jss.v023.i04 (2007).

[25] R Core Team. *R: A Language and Environment for Statistical Computing*. (R Foundation for Statistical Computing, 2020).

[26] Nelson, A. S., Weiskittel, A. R., Wagner, R. G. & Saunders, M. R. Development and evaluation of aboveground small tree biomass models for naturally regenerated and planted species in eastern Maine, U.S.A. *Biomass Bioenergy* **68**, 215–227, 10.1016/j.biombioe.2014.06.015 (2014).

[27] Poudel, K. P., Temesgen, H., Radtke, P. J. & Gray, A. N. Estimating individual-tree aboveground biomass of tree species in the western U.S.A. *Can. J. For. Res.* **49**, 701–714, 10.1139/cjfr-2018-0361 (2019).

[28] Carvalho, J. P. & Parresol, B. R. Additivity in tree biomass components of Pyrenean oak (*Quercus pyrenaica* Willd.). *For. Ecol. Manag.* **179**, 269–276, 10.1016/S0378-1127(02)00549-2 (2003).

[29] He H (2018). Allometric biomass equations for 12 tree species in coniferous and broadleaved mixed forests, Northeastern China. PLoS ONE.

[30] Cheng C-H, Huang Y-H, Menyailo OV, Chen C-T (2016). Stand development and aboveground biomass carbon accumulation with cropland afforestation in Taiwan. Taiwan J. For. Sci..

[31] Lee J-H, Ko Y, McPherson EG (2016). The feasibility of remotely sensed data to estimate urban tree dimensions and biomass. Urban For. Urban Green..

[32] Park JH, Baek SG, Kwon MY, Je SM, Woo SY (2018). Volumetric equation development and carbon storage estimation of urban forest in Daejeon, Korea. For. Sci. Technol..

[33] Yoon TK (2013). Allometric equations for estimating the aboveground volume of five common urban street tree species in Daegu, Korea. Urban For. Urban Green..

[34] Chiu, C. M., Lo-Cho, C.-N. & Suen, M.-Y. Pruning method and knot wound analysis of Taiwan zelkova (*Zelkova serrata* Hay.) plantations. *Taiwan J. For. Sci.* **17**, 503–513, 10.7075/TJFS.200212.0503 (2002).

[35] Lo-Cho, C.-N., Chung, H.-H. & Chiu, C.-M. Effects of pruning on the growth and the branch occlusion tendency of Taiwan Zelkova (*Zelkova serrata* Hay.) young plantations. *Bull. Taiwan For. Res. Inst.* **10**, 315–323, 10.7075/BTFRI.199509.0315 (1995).

[36] Shepherd KR (1986). Plantation Silviculture.

[37] Chiou C-R, Lin J-C, Liu W-Y (2019). The carbon benefit of thinned wood for bioenergy in Taiwan. Forests.

[38] Liu W-Y, Lin C-C, Su K-H (2017). Modelling the spatial forest-thinning planning problem considering carbon sequestration and emissions. For. Policy Econ..

[39] Rais A, Poschenrieder W, van de Kuilen J-WG, Pretzsch H (2020). Impact of spacing and pruning on quantity, quality and economics of Douglas-fir sawn timber: Scenario and sensitivity analysis. Eur. J. For. Res..

[40] Kershaw, J. A., Ducey, M. J., Beers, T. W. & Husch, B. *Forest Mensuration*. (John Wiley & Sons Ltd, 2016).

